# Promises and pitfalls of Web-based experimentation in the advance of replicable psychological science: A reply to Plant (2015)

**DOI:** 10.3758/s13428-015-0677-x

**Published:** 2015-11-05

**Authors:** Henk van Steenbergen, Bruno R. Bocanegra

**Affiliations:** 1 Institute of Psychology, Leiden University, Leiden, The Netherlands; 2 Wassenaarseweg 52, 2333 AK Leiden, The Netherlands; 3 Leiden Institute for Brain and Cognition, Leiden University, Leiden, The Netherlands

**Keywords:** Online experimentation, Web-based psychology experiments, Psychological science, Replication, Qualtrics Reaction Time Engine (QRTEngine), Critical timing, Reaction times

## Abstract

In a recent letter, Plant (2015) reminded us that proper calibration of our laboratory experiments is important for the progress of psychological science. Therefore, carefully controlled laboratory studies are argued to be preferred over Web-based experimentation, in which timing is usually more imprecise. Here we argue that there are many situations in which the timing of Web-based experimentation is acceptable and that online experimentation provides a very useful and promising complementary toolbox to available lab-based approaches. We discuss examples in which stimulus calibration or calibration against response criteria is necessary and situations in which this is not critical. We also discuss how online labor markets, such as Amazon’s Mechanical Turk, allow researchers to acquire data in more diverse populations and to test theories along more psychological dimensions. Recent methodological advances that have produced more accurate browser-based stimulus presentation are also discussed. In our view, online experimentation is one of the most promising avenues to advance replicable psychological science in the near future.

In a recent letter, Plant (2015) reminded us about the importance of benchmarking our psychological paradigms. New hardware and software developments often tend to make the timing of experiments more imprecise, and Plant argues that this might be a locus for the failure to replicate earlier published findings, a concern of primary importance these days in psychology and other field of science (Ioannidis, 2005; Pashler & Wagenmakers, 2012; Stroebe & Strack, 2014). Following this line of reasoning, Plant argues that carefully controlled laboratory experiments should be preferred over more imprecise methods available for Web-based experimentation, because the former are more amenable to controlled external stimulus calibration. Although we agree with Plant that the role of stimulus calibration has been underappreciated with regard to the replicability of findings (see Plant, 2015), we do not believe that the inherent imprecision of Web-based experimentation precludes it from becoming a promising new tool in the psychologist’s toolbox, alongside lab-based approaches.

Plant’s letter raises a number of important questions, such as: Against what should we calibrate an experimental setup in psychology, and when is stimulus calibration necessary in an experimental setup in psychology? After reading Plant’s letter, the reader might get the impression that any experiment that manipulates stimulus presentation time should always be calibrated against an external stimulus criterion. However, as Plant admits himself, there are cases in which timing inaccuracy may be acceptable, especially if the method does not for call for a high degree of precision. Here we will highlight these situations and argue that Web-based experimentation provides many research opportunities that are not possible with standard lab experimentation, making this is an interesting avenue for future exploration.

We agree with Plant that when experimental findings depend on precise timing or presentation, calibrating to a stimulus criterion is essential. When utilizing complex cognitive paradigms and neuroimaging techniques, continuous external chronometry (e.g., Plant, Hammond, & Turner, 2004) and the use of special hardware (e.g., the new device Chronos, developed by Psychology Software Tools, Inc.) might indeed be the only way to guarantee accurate timing and draw correct conclusions. For example, in a cognitive experiment in which an auditory tone is presented as an accessory stimulus in combination with a Stroop task, timing accuracy is important, since the timing characteristics may affect the size of the impact of the accessory stimulus on the conflict task. Also, if a researcher’s claim depends on knowing the absolute duration or specific physical parameters of a stimulus (e.g., when determining the temporal response profiles or receptive field properties of neurons), then it is imperative to calibrate against a stimulus criterion.

However, for an average psychological experiment this is not always as pertinent. Let’s take the standard Stroop task as an example. This task is used by a wide range of researchers, from domains as diverse as cognitive neuroscience, social psychology, and neuropsychology. Is it critical that the timing of the stimulus duration and response registration in this task have millisecond accuracy? Likely not. The Stroop effect was originally assessed on a piece of paper, and some modern neuropsychological tests still assess it using paper and stopwatch only. Although stopwatch accuracy is certainly not ideal (see Doyen, Klein, Pichon, & Cleeremans, 2012), in some cases it is sufficient. Similarly, when using inaccurate computer-based timing, enough observations will likely yield a reliable behavioral measure, as long as the measurement error is random and nonsystematic. The situation is comparable to other types of calibration, such as color (Metha, Vingrys, & Badcock, 1993). Monitors that present the Stroop word “red” in a red font are likely to display different shades of red. The more the color red deviates from a standard red hue, the smaller will be the interference effect the word produced, which may invalidate the interpretation of the results. However, researchers usually do not worry about calibrating their setup to a stimulus criterion when designing a Stroop task, because the error variance present is likely to be quite small. Rather, researchers using Stroop tasks are more interested to check whether participants are able to successfully differentiate and identify the different color stimuli, despite there being some stimulus variability. In other words, they calibrate their setup to a response criterion.

Even in situations in which researchers are interested in elementary visual processing, such as when using a masked priming experiment, calibration to stimulus criteria may not always be necessary. On the other hand, calibration to appropriate response criteria seems essential in this case. For example, a researcher may want to present stimulus durations in such a way that participants can reliably identify a stimulus in a visible condition and fail to identify it in a masked condition (see Sumner, Tsai, Yu, & Nachev, 2006), or perhaps a researcher needs to establish that reaction times increase linearly as a function of onset latency (Vorberg, Mattler, Heinecke, Schmidt, & Schwarzbach, 2003).

For example, let’s say Researcher A sets up a priming experiment that he calibrates so as to ensure a prime presentation of 30 ms using external chronometry. He also calibrates according to a response criterion: The stimulus duration is such that participants are at chance-level accuracy in identifying the prime stimulus. His colleague, Researcher B, attempts a replication using the same hardware and software. She calibrates the setup to the desired 30-ms stimulus presentation time, again validated by external chronometry. Surprisingly, however, she observes a very different pattern of results than her colleague Researcher A. How could this be, given that she presented exactly the same stimulus parameters? She decides to do a control experiment. Here she observes that her participants are above chance at identifying the prime, and under those conditions the observed pattern of results becomes readily interpretable. Effects such as these are not merely hypothetical. It is well known that under identical experimental conditions, masked priming effects may vary considerably as a function of individual differences: for example, differences in masking efficiency (Eimer & Schlaghecken, 2002), age (Schlaghecken & Maylor, 2005), handedness (Serrien, Sovijärvi-Spapé, & Rana, 2012), and video-gaming experience (Pohl et al., 2014).

In practice, calibration to response criteria is usually done by first performing pilot studies in order to set the experimental parameters. Subsequently, this calibrated setup is used to test a new hypothesis. In this way, one is in effect setting a baseline against which to design and interpret methodological extensions. There are many examples in experimental psychology in which, in order to replicate an empirical phenomenon, one first has to calibrate one’s stimuli so as to elicit a certain pattern of behavioral responses. For example, in order to observe a negative compatibility or conflict adaptation effect, one’s stimuli have to be able to elicit response conflict (Eimer & Schlaghecken, 2002; Gratton, Coles, & Donchin, 1992). In order to observe an emotion-induced blindness effect, one’s stimuli have to elicit differences in arousal judgments (Most, Chun, Widders, & Zald, 2005). In order to observe lag-1 sparing, one’s stimulus sequence has to be able to elicit an attentional blink (Hommel & Akyürek, 2005). Calibration to response criteria is an integral part of setting up experiments in psychology. However, Plant (2015) seems to disagree:

Worryingly in the literature there seems to be a growing trend toward “checking” timing accuracy by proxy by running a study and if the results generally tally with those expected or are in line with previously published findings then the timing “must” have been acceptable. Indeed, this is how the authors of QRTEngine, an online experiment generator and delivery system . . . , validated its efficacy rather than using external chronometry to check presentation and response timings. In no other field of science would this be viewed as acceptable. (p. 3)

We are afraid that Plant has misinterpreted our point. We do not suggest that by observing an experimental effect one can check whether the actual stimulus presentation within one’s own setup had a certain value (say, 16 ms). Indeed, external chronometry should be used to support claims about stimulus duration, and—note—this is indeed what we did and report by running an experiment that measured the duration of black squares presented on a white background using a photosensitive diode attached to the monitor. So, Plant here clearly misrepresented the method we used to validate the Qualtrics Reaction Time Engine (QRTEngine). On the other hand, Plant is right, in the sense that we did not validate the response timings of the QRTEngine. We hesitated to do so because the participants in Web experiments will use different types of response devices that, simply due to the fact that keyboard hardware is typically not optimized for high-precision timing, will produce uncontrollable and considerable measurement errors (e.g., Plant & Turner, 2009). Moreover, even when using external chronometry, it is impossible to dissociate the accuracy of a response device itself from the inaccuracies in the software method to register response onset. However, there is currently no reason to assume that the accuracy of response times assessed with JavaScript-based methods such as those used in the QRTEngine is worse than the accuracy of procedures used in other software packages. Indeed, a recent study that directly compared JavaScript response collection accuracy to the MATLAB Psychophysics Toolbox did not observe a reliable difference in the variability of the response time distributions between these two packages, suggesting that JavaScript is suitable for reliable response time measurements (de Leeuw & Motz, 2015).

In any case, Plant seems to underappreciate the fact that benchmarking an experimental setup in psychology involves more than just making sure that stimuli are accurately and reliably presented. The reason why we ran a set of experiments was to show that known behavioral signatures such as masked priming, response conflict, and the attentional blink can be observed online (for a similar approach, see Crump, McDonnell, & Gureckis, 2013). In general, the practice of calibration against response criteria is very important in psychology, and we believe it quite unfortunate that Plant insinuates that doing so would not be acceptable practice in other scientific disciplines. In fact, this statement is also factually inaccurate: Experiments on animal behavior routinely calibrate to response criteria, precisely because the stimulus parameters underlying manipulations often exert their effects on organisms’ behavior in context-sensitive ways and in ways that depend on the intrinsic characteristics of the organism (Lehner, 1998; Martin & Bateson, 1993).

In experimental ethology, for example, it has been known ever since the seminal work of Nico Tinbergen (1951) that it is not so much the calibration of stimuli to identical physical criteria that enables researchers to replicate each other’s findings, but rather the calibration of stimuli to the response criteria of the organisms. Importantly, given that animal and human behavioral responses to stimuli depend, among other factors, on circadian, social, ontogenetic, motivational, and intrinsic factors, calibrating to response criteria is imperative to replicating findings in any behavioral science (see chapter 2 in Tinbergen, 1951, for many beautiful examples). Not only is calibrating to response criteria good practice in psychology, but it may also be key to improving the replicability of findings, precisely because, unlike in physics, experimental effects in psychology depend on more than just hardware and software: They depend on the context that participants find themselves in and on their intrinsic characteristics.

It is for this reason that we think Web-based experimentation may be very helpful. Doing research in a lab is inherently constraining in terms of the participants’ characteristics and the contextual variables that can be systematically investigated. Although differences in timing accuracy may indeed account for failures to replicate complicated experiments in some disciplines, we think that in the broader field of psychology small sample sizes and banal and idiosyncratic differences in lab contexts (e.g., different fonts used; see Jolicœur, Snow, & Murray, 1987), as well as the presence of unknown contextual and intrinsic moderating factors, are much more serious reasons for concern. Indeed, this is the motivation for recent replication projects that include replications over many labs (Open Science Collaboration, 2012; for recent examples, see Alogna et al., 2014; Klein et al., 2015; Open Science Collaboration, 2015). We envision that the use of online labor markets, such as Amazon’s Mechanical Turk, a relatively inexpensive method to test hundreds of participants in a couple of hours, will help to bring progress to many psychological subdisciplines using paradigms that do not critically depend on millisecond accuracy and supervised assessment (cf. Buhrmester, Kwang, & Gosling, 2011; Crump et al., 2013; Mason & Suri, 2012). As a useful side effect, using online tasks in open formats that run on different platforms will likely also facilitate transparency and quicker replication by fellow researchers.

Online testing and big-data analytic approaches will likely lead to dramatic changes in the ways that we conduct science over the next two or so decades (cf. Griffiths, 2015). Such directions are currently already being successfully pursued using social media data (e.g., Eichstaedt et al., 2015; Park et al., 2015), and we expect that they will soon also start to shape the field of psychology as a whole. Online testing comes with many opportunities (see also Crump et al., 2013; Griffiths, 2015; Mason & Suri, 2012). For example, it will allow us to test hypotheses within a much larger domain of potentially interesting psychological dimensions, thus allowing us to draw landscapes of generalizability (Brunswik, 1955), instead of the traditional approach of testing a small convenience sample and merely hoping that the effects will generalize to different populations (cf. Asendorpf & Conner, 2012). Moreover, time-consuming laboratory experiments that include many repetitions and a few subjects may be complemented by one-shot online experiments that include thousands of participants (e.g., Paolacci, Chandler, & Ipeirotis, 2010), allowing researchers to answer new questions in novel ways that were not possible using traditional means of data acquisition. Given the short empirical cycle enabled by online studies, Internet-based experiments may also be used to pilot new paradigms before they are tested extensively in the lab, saving researchers’ time and facilitating scientific progress.

Although methodological challenges will need to be addressed in future optimizations, an increasing number of studies indicate that the timing characteristics of browser-based experiments are acceptable enough to warrant the replication of default laboratory experiments (Barnhoorn, Haasnoot, Bocanegra, & van Steenbergen, 2015; Chetverikov & Upravitelev, 2015; de Leeuw, 2015; de Leeuw & Motz, 2015; Garaizar, Vadillo, & López-de-Ipiña, 2014; Keller, Gunasekharan, Mayo, & Corley, 2009; Neath, Earle, Hallett, & Surprenant, 2011; Reimers & Stewart, 2015; Schubert, Murteira, Collins, & Lopes, 2013; Slote & Strand, 2015; von Bastian, Locher, & Ruflin, 2013). In particular, the introduction of HTML5 presentation techniques has enabled frame-wise stimulus presentation for browser-based experiments, a method we have included in the QRTEngine (Barnhoorn et al., 2015), which allows millisecond accuracy to be approached (Garaizar et al., 2014). We also foresee that future versions of Qualtrics will enable researchers to preload surveys (experiments) in a browser’s cache, which will contribute toward achieving full control over the timing of the intertrial intervals in our QRTEngine tool. Although we fully agree with Plant that Web-based experimentation should be used with care and deliberation (see also Barnhoorn et al., 2015), there are quite a few reasons to be optimistic. We believe that for many psychological paradigms, online experimentation is one of the most promising avenues to advance replicable psychological science in the future.
